# Coaxial Double-Pigtail Stent Placement: A Simple Solution to Decrease Bleeding Risk Associated With Lumen-Apposing Metal Stent?

**DOI:** 10.7759/cureus.15981

**Published:** 2021-06-28

**Authors:** Shehroz Aslam, Zaid Ansari, Mustafa Alani, Indu Srinivasan, Keng-Yu Chuang

**Affiliations:** 1 Internal Medicine, Creighton University School of Medicine, St. Joseph's Hospital and Medical Center, Phoenix, USA; 2 Gastroenterology, Creighton University School of Medicine, St. Joseph's Hospital and Medical Center, Phoenix, USA; 3 Gastroenterology, Valleywise Health Medical Center, Phoenix, USA; 4 Gastroenterology, Creighton University School of Medicine, Phoenix, USA

**Keywords:** pancreatic fluid collections, cystogastrostomy, gastrointestinal hemorrhage, pseudoaneurysm, lams

## Abstract

Endoscopic cystogastrostomy using lumen-apposing metal stent (LAMS) is considered the first-line therapy for symptomatic pancreatic fluid collections (PFCs). Routine coaxial placement of a double-pigtail stent (DPS) through LAMS is debated. We report the case of a patient with delayed massive gastrointestinal bleed eight weeks after LAMS placement due to splenic artery pseudoaneurysm leading to a complicated hospitalization. Theoretically, coaxial placement of DPS through LAMS can prevent the relatively sharp LAMS from eroding into the mucosa of the collapsed cavity of PFCs, decreasing the risk of bleeding. Our case adds to the growing need to further explore the utility of this combined intervention.

## Introduction

Pancreatic fluid collections (PFCs) are frequent complications of acute as well as chronic pancreatitis and include pancreatic pseudocyst and walled-off necrosis (WON) [1]. Most of the PFCs resolve spontaneously; however, in some cases, when left untreated they can increase in size resulting in persistent abdominal pain, infection, and biliary obstruction [2]. In these cases, non-surgical interventions for cyst drainage are preferred over surgical procedures. Cystogastrostomy through endoscopic ultrasound (EUS) has gained significant importance in the management of symptomatic pancreatic pseudocyst and WON [3]. The use of lumen-apposing metal stent (LAMS) and double-pigtail stent (DPS) has been studied for this procedure. Before the development of LAMS, DPSs were extensively used but their narrow diameters limit the drainage of solid necrotic debris; this limitation was overcome with the larger-diameter LAMS. Additionally, LAMS allows endoscopic necrosectomy through the stent lumen. LAMS, however, is associated with bleeding and stent migration [4,5]. Coaxial placement of DPS through LAMS has been mentioned in the literature with a goal to reduce the bleeding risk but outcome data remain sparse. We report a case of an elderly male who presented to our facility with a massive gastrointestinal (GI) bleed following LAMS placement for WON and found to have a splenic artery pseudoaneurysm requiring surgical intervention.

## Case presentation

**Figure 1:**
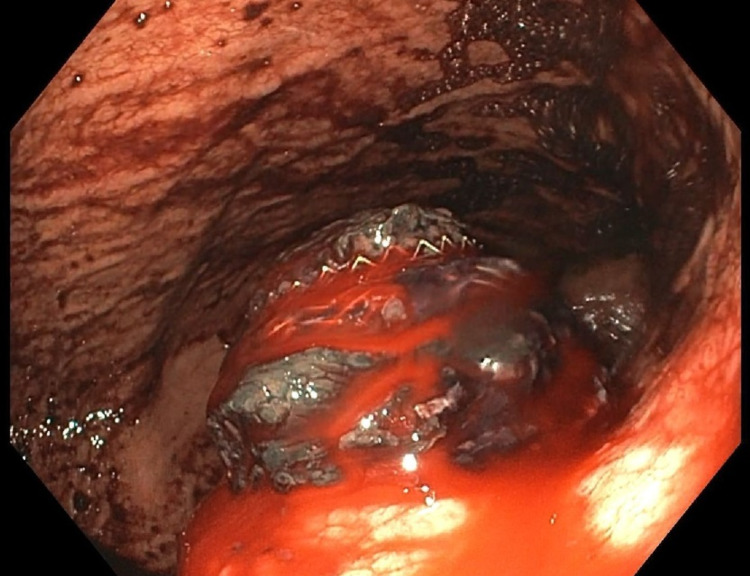
Slow oozing of blood noted around the stent

**Figure 2:**
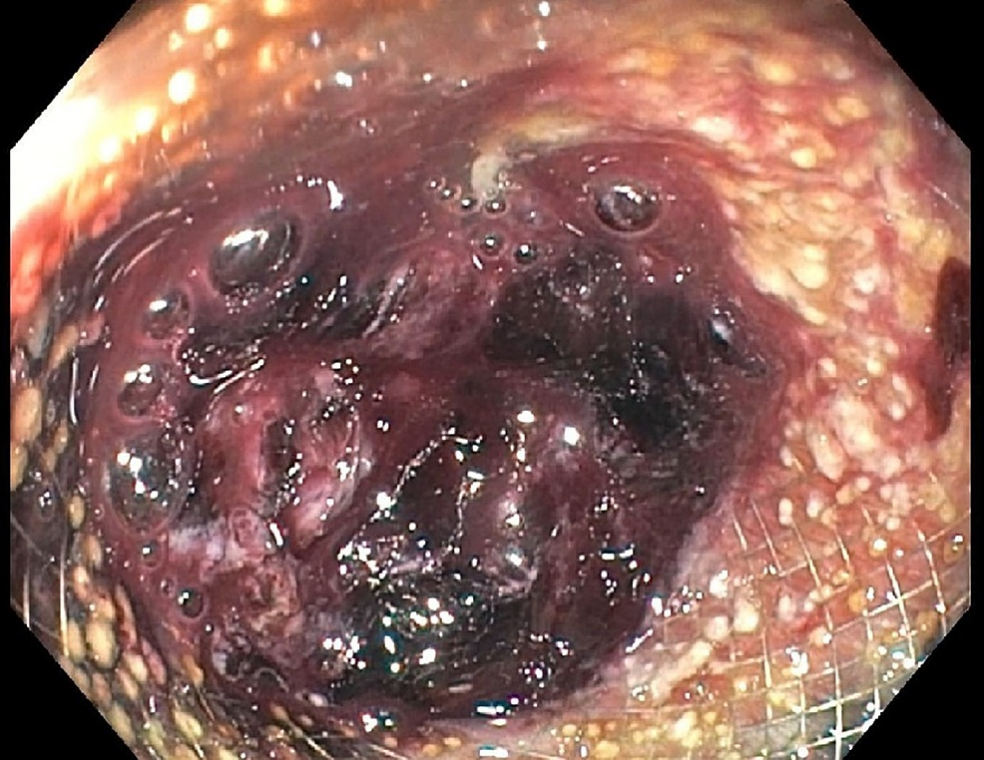
Second-look endoscopy revealing blood clots in the stent with no active bleeding

**Figure 3:**
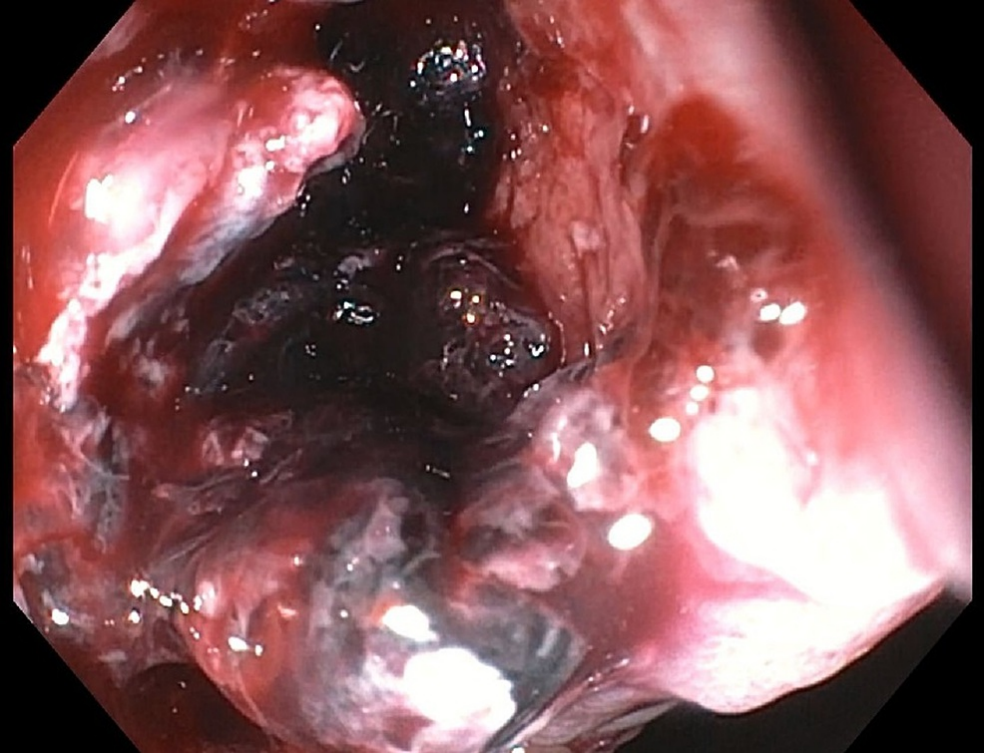
Stent removed and revealed no active bleeding within the cystogastrostomy cavity

A 72-year-old male with a history of chronic pancreatitis and WON (measuring 6 x 4 x 5 cm) presented to our facility with hematemesis. Eight weeks before the presentation, he underwent cystogastrostomy with necrosectomy and LAMS (AXIOS^TM^, Boston Scientific, MA, USA) placement at an outside hospital. On presentation, his vital signs were stable with a normal physical examination. His hemoglobin (Hgb) was 9.3 gram/deciliter (g/dL), down from his baseline of 11 g/dL. Esophagogastroduodenoscopy (EGD) was performed, which showed slow oozing from the cystogastrostomy site and a large blood clot within the LAMS (Figure 1). A computed tomography angiography (CTA) performed did not demonstrate bleeding near the cystogastrostomy site but active contrast extravasation into the rectum via the inferior mesenteric artery (IMA) was noted. A branch of the IMA was then super-selectively embolized by interventional radiology (IR). The next day, patient had a 2-g drop in Hgb and a second-look EGD showed a stable clot within the LAMS without bleeding, the stent was removed, and the cavity was inspected carefully. No stigmata of recent bleed were seen within the cavity (Figures 2, 3). A colonoscopy also was performed, which was unremarkable. Roughly 1 h after the procedure, the patient had massive hematemesis resulting in hemorrhagic shock. A third EGD showed active bleeding from the cystogastrostomy site with very limited endoscopic visualization. The patient was taken for an exploratory laparotomy by general surgery where a small incidental gastric perforation was identified in the gastric body near the cystogastrostomy site. The perforation was repaired along with surgical closure of the cystogastrostomy. Post-operatively the patient had a good recovery and was discharged. 

**Figure 4:**
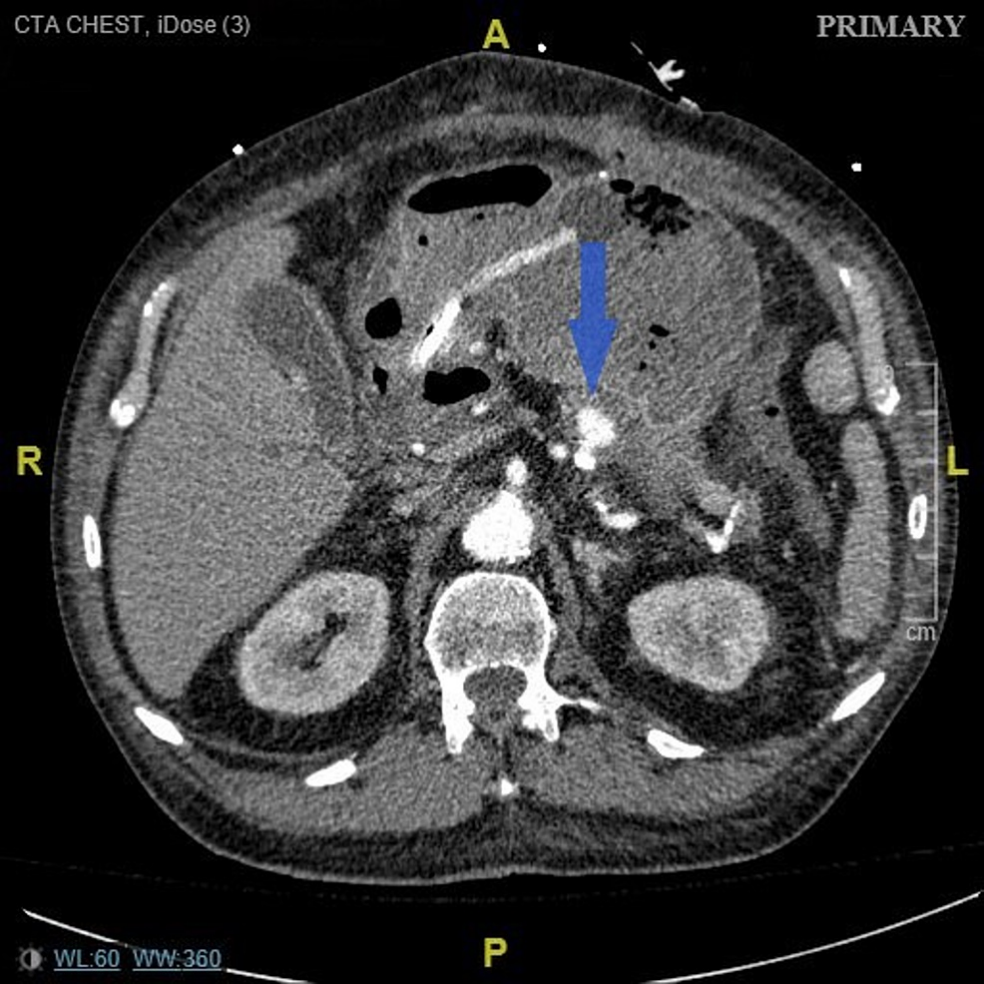
Computed tomography angiography showing splenic artery pseudoaneurysm measuring 3.6 x 1.8 x 1.4 cm (blue arrow)

**Figure 5:**
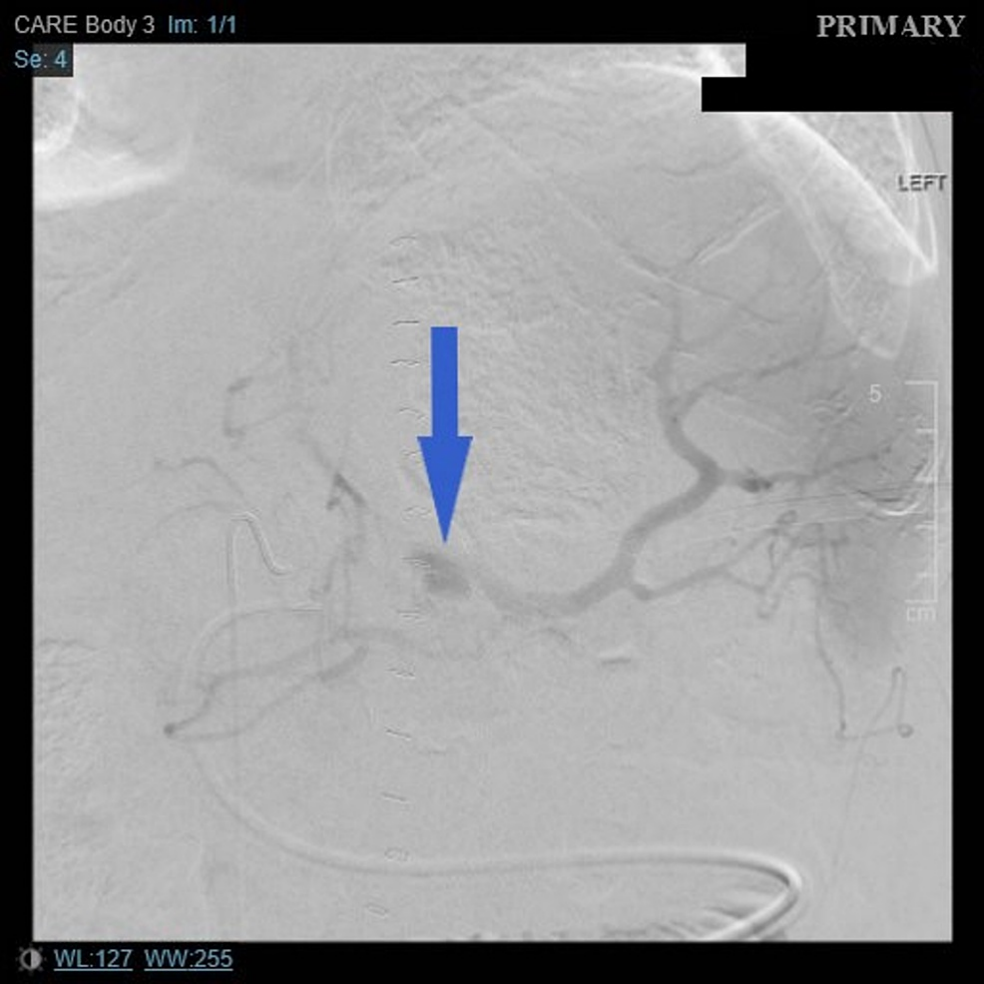
Interventional radiology image of mid splenic artery pseudoaneurysm (blue arrow)

Three days later, he returned to the hospital with another episode of hematemesis with hemodynamic instability. CTA was again obtained, and this time a splenic artery pseudoaneurysm (3.6 x 1.8 x 1.4 cm) with active bleeding was identified, which was embolized by IR (Figures 4, 5). He, however, had another massive hematemesis 72 h later. A bedside EGD was attempted but failed to achieve hemostasis due to poor visualization. He was taken for another exploratory laparotomy, which revealed active bleed from the splenic artery pseudoaneurysm. This was managed with surgical ligation of the splenic artery, splenectomy, and partial gastrectomy. Reconstructive Roux-en-Y gastrojejunostomy was performed a week later.

 

Post-operatively the patient had a protracted hospital course on his way to recovery. No further GI bleeding was noted during the remaining hospital stay. He was eventually discharged home with close outpatient follow-up. 

## Discussion

EUS-guided cystogastrostomy has emerged as the gold standard for the management of PFCs [6,7]. Plastic stents in the double-pigtail configuration were initially preferred as they prevented migration; their narrow lumen, however, can lead to premature stent occlusion in up to 18% of cases, resulting in frequent stent exchanges or placement of additional stents [8,9]. Recently developed LAMS in the “dumbbell” configuration with two large flanges can minimize stent migration. The larger diameter of LAMS also allows endoscopic necrosectomy in repeated sessions without the need for stent replacement [4].

In a review, Patil et al. reported a 97% technical success rate and a 96% clinical success in 298 patients undergoing EUS-guided LAMS placement [10]. While LAMS and DPS have been widely studied individually, head-to-head comparisons of the two modalities are limited: an interim analysis of a randomized controlled trial by Bang et al. reported serious adverse events three weeks after the index intervention where three out of the 12 patients randomized for LAMS presented with severe GI bleeding due to a pseudoaneurysm [11]. Brimhall et al. in a retrospective analysis demonstrated comparable rates of technical and clinical success; the use of LAMS, however, was associated with a higher bleeding risk from pseudoaneurysm compared to DPS (odds ratio, 10.0; 95% confidence interval, 1.19-84.6; P = 0.009) [12].

**Figure 6:**
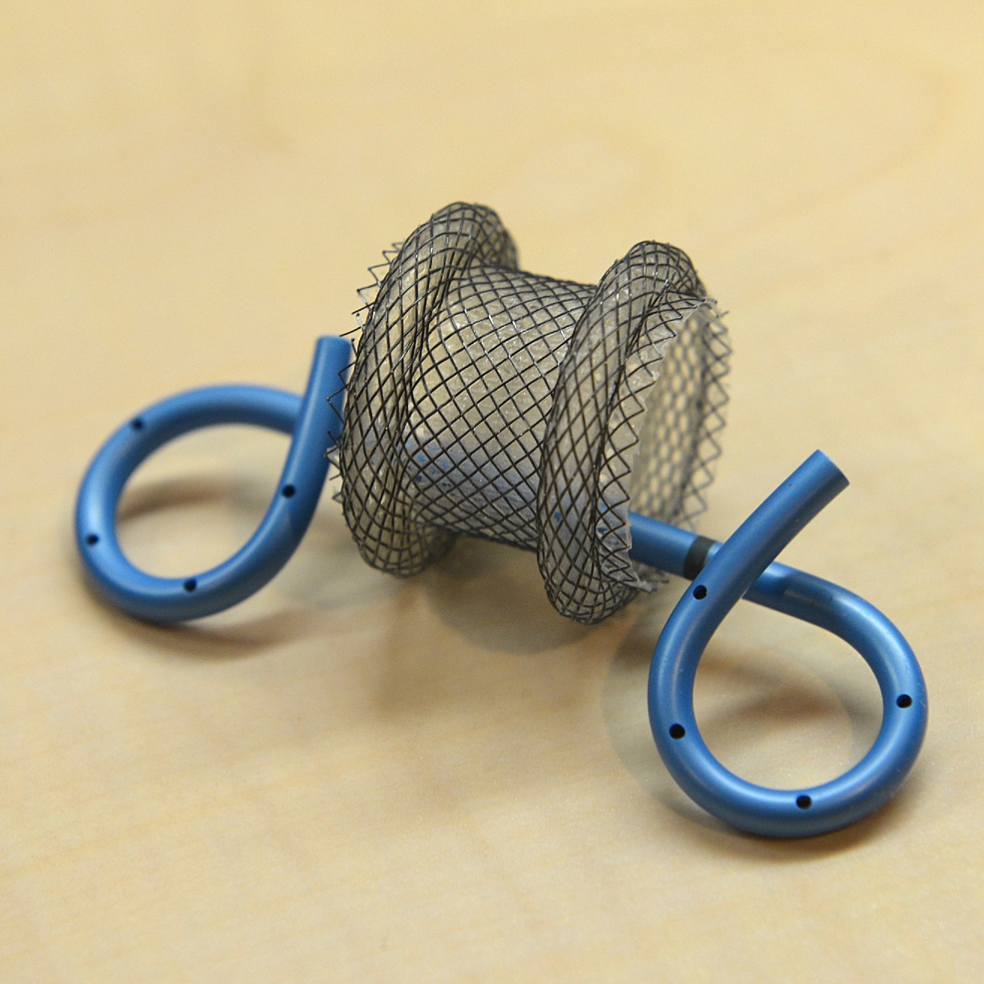
Coaxial placement of double-pigtail stent through lumen-apposing metal stent (AXIOS)

In recent years, the use of coaxial placement of DPS through LAMS to decrease bleeding complications has gained popularity (Figure 6). The placement of DPS through LAMS helps to prevent direct contact of the mucosa and surrounding vessels with the relatively sharp flange of LAMS following the collapse of PFCs. A pilot study by Aburajab et al. reported a higher risk of infection and non-resolution of pancreatic pseudocyst with the use of LAMS alone. The risk, however, was decreased with the placement of DPS across LAMS (relative risk, 0.84; 95% confidence interval, 0.71-1.0; P = 0.054) [13]. Another retrospective study by Puga et al. reported that LAMS alone had a significantly higher rate of adverse events compared to LAMS plus coaxial DPS for drainage of PFCs (42.9% vs 10.0%; P = 0.04) with bleeding being the most common adverse event [14]. Rossi et al. analyzed the efficacy of coaxial DPS placement into LAMS with a secondary aim to decrease the bleeding risk. The bleeding rate in patients with DPS was lower (5.9%) compared to those without (15.6%) even though the difference did not achieve statistical significance (P = 0.65) [15].

The combined use of DPS and LAMS is an ongoing topic of research. NCT03923686 is a prospective randomized trial, which is underway, analyzing the coaxial placement of DPS through LAMS for EUS-guided transmural drainage of WON [16]. The study one day might guide us to a safer use of LAMS in patients with PFCs.

## Conclusions

The coaxial placement of DPS through LAMS to prevent bleeding risk remains a subject of discussion in the field of gastroenterology. We discussed a case of an elderly gentleman who presented with massive delayed splenic artery pseudoaneurysm bleeding following placement of LAMS for WON, a known complication of LAMS. This complication might have been prevented with the coaxial placement of DPS through LAMS. Based on our case we advocate for the routine use of DPS through LAMS to mitigate bleeding risks. At the same time, we encourage future randomized trials to further analyze this combined intervention.
